# Brain imaging reveals covert consciousness during behavioral unresponsiveness induced by propofol

**DOI:** 10.1038/s41598-018-31436-z

**Published:** 2018-09-04

**Authors:** Zirui Huang, Phillip E. Vlisides, Vijaykumar C. Tarnal, Ellen L. Janke, Kelley M. Keefe, Margaret M. Collins, Amy M. McKinney, Paul Picton, Richard E. Harris, George A. Mashour, Anthony G. Hudetz

**Affiliations:** 1 Department of Anesthesiology, University of Michigan Medical School, Ann Arbor, MI USA; 2 Center for Consciousness Science, University of Michigan Medical School, Ann Arbor, MI USA; 3 Neuroscience Graduate Program, University of Michigan, Ann Arbor, MI USA

## Abstract

Detecting covert consciousness in behaviorally unresponsive patients by brain imaging is of great interest, but a reproducible model and evidence from independent sources is still lacking. Here we demonstrate the possibility of using general anesthetics in a within-subjects study design to test methods or statistical paradigms of assessing covert consciousness. Using noninvasive neuroimaging in healthy volunteers, we identified a healthy study participant who was able to exhibit the specific fMRI signatures of volitional mental imagery while behaviorally unresponsive due to sedation with propofol. Our findings reveal a novel model that may accelerate the development of new approaches to reproducibly detect covert consciousness, which is difficult to achieve in patients with heterogeneous and sometimes clinically unstable neuropathology.

## Introduction

Covert consciousness is defined as the presence of subjective experience in the absence of behavioral response. Despite the successful administration of general anesthesia in hundreds of millions of patients around the globe each year, covert consciousness with explicit recall occurs in 0.15% of surgical cases^1,2^, and, conservatively, in approximately 5% of surgical cases without explicit recall^3^. Covert consciousness can occur during surgery due to the administration of neuromuscular blockers, which prevent behavioral response to noxious stimuli. Similarly, in patients with pathologic disorders of consciousness, such as vegetative state/unresponsive wakefulness syndrome (VS/UWS)^4,5^, covert consciousness can occur without any overt behavioral signs by standard clinical assessment^6,7^. Therefore, a reproducible brain signature of consciousness independent of behavior would greatly enhance its accurate determination.

In 2006, it was reported that a patient with a diagnosis of VS/UWS evidenced signs of consciousness and volitional response, as detected through a neuroimaging paradigm^6^. With the use of functional magnetic resonance imaging (fMRI), the investigators were able to identify activity of the supplementary motor area (SMA) after asking the patient to imagine playing tennis as well as activity of the parahippocampal place area (PPA) and posterior parietal cortex (PPC) after asking the patient to imagine walking through their home. This striking pattern of activity was identical to that exhibited by healthy controls. There have been further studies using fMRI mental imagery paradigms in patients with disorders of consciousness^7–10^. More recently, successful command-following was demonstrated in behaviorally unresponsive patients diagnosed with VS/UWS using EEG-based signatures that, in some cases, were paralleled by corresponding fMRI signatures of imagery task performance^11^. Taken together, these results suggest that a fMRI mental imagery paradigm can demonstrate consciousness, where awareness is defined as intentional neural modulation or neural “command following”^12^.

Nevertheless, the approach of investigating covert consciousness in patients with disorders of consciousness has disadvantages. The putative signatures of covert cognition in patients with differing neurological injuries can be limited by considerable inter-individual variability^11^. Another limitation of such studies is the restriction to a between-subjects design, in which patients are compared to healthy controls. This is potentially problematic because the functional areas mediating imagery tasks may be different in patients and in healthy controls. Moreover, many VS/UWS patients have gross structural abnormalities including, or close to, the imagery task-related regions (e.g. SMA), confounding the determination of relevant brain activations. Finally, we also lack a precise neural account for the paradoxical impairment of purposeful motor behavior in covertly aware patients^13–15^. A previous study examined the importance of selective structural damage to thalamocortical motor fibers as an explanation for the lack of intentional movement in a single VS/UWS patient with identified covert consciousness^16^. Thus, whether structural damage is a necessary condition for the dissociation between covert (i.e. non-behavioral) consciousness and overt (i.e. behavioral) responsiveness is yet unsettled.

To-date, there is no consensus regarding the objective, measureable signatures of human consciousness. Hence, a reproducible model and a broad range of evidence from independent sources can be of great value to further validate the brain imaging approach in detecting covert consciousness^9,10,15,17–19^. Testing the hypothesis of covert consciousness with anesthetic drugs offers the possibility of within-subject design, minimizing the variability across subjects, and is not confounded by unpredictable structural lesions that may preclude behavioral response^17^. With this approach, the same subject can be evaluated during the same task at precisely controlled levels of consciousness, including wakefulness and anesthetized states induced by appropriate drug dosing.

We hypothesized that we could reproduce the formerly observed dissociation of consciousness and behavioral responsiveness using noninvasive neuroimaging of healthy volunteers at carefully controlled anesthetic levels. By titrating the anesthetic dose to achieve behavioral unresponsiveness and using rigorous false positive control, we report a case of such dissociation.

## Results

### Functional MRI paradigm for testing covert consciousness in a pharmacological setting

**Figure 1:**
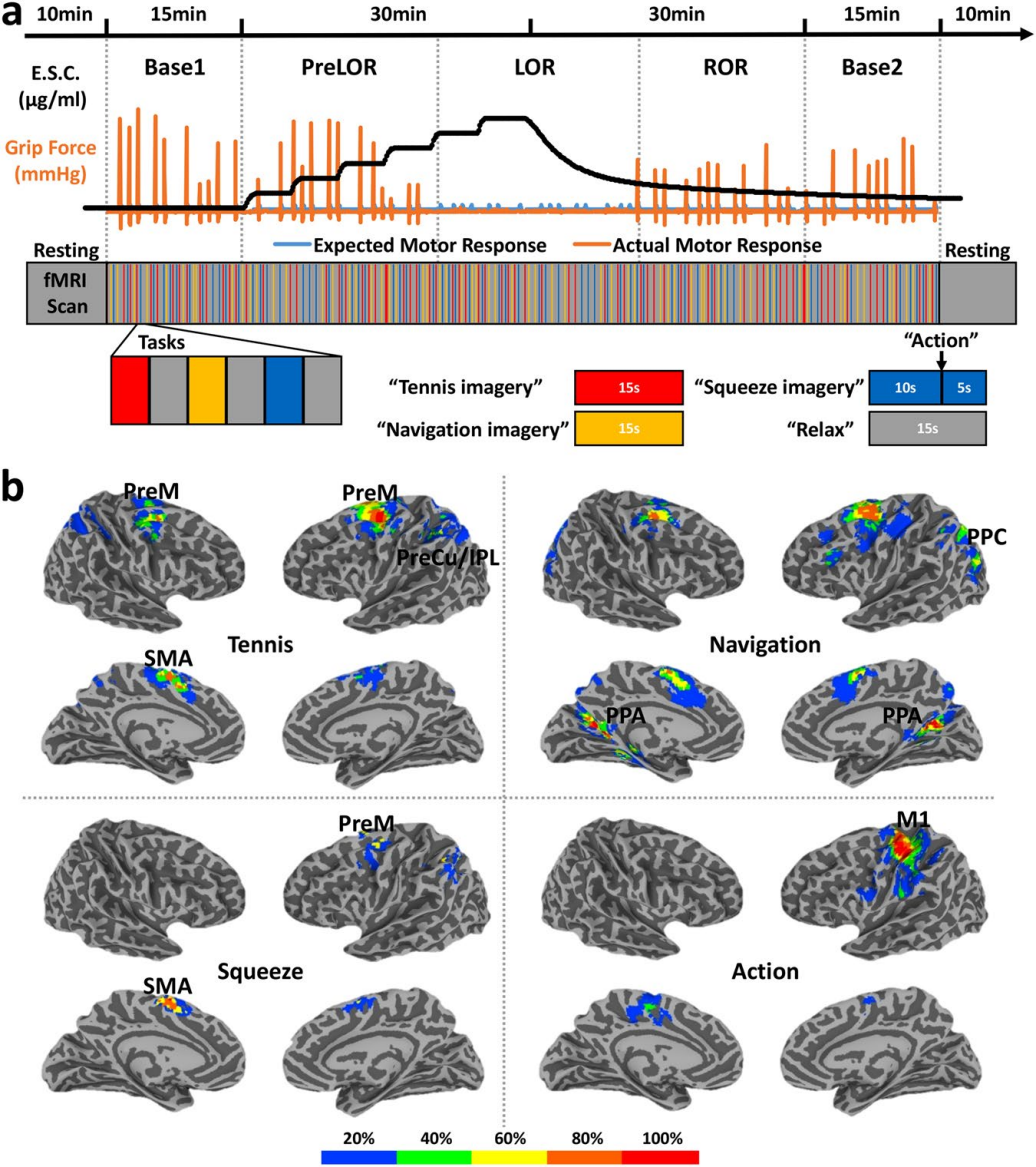
Experimental design and regions of interest (ROIs). (**a**) Schematic of experimental set-up with stepwise intravenous infusion of propofol and fMRI tasks. The infusion rate was controlled to achieve propofol effect-site concentrations (E.S.C.) of 0, 0.4, 0.8, 1.2, 1.6, 2.0, and 2.4 μg/ml and each level was maintained for 5 minutes. Emergence from sedation began at the end of the 2.4 μg/ml test period by stopping the infusion. Mental imagery and motor response tasks were tested before, during and after propofol infusion. Subjects were asked to perform three imagery tasks (tennis, navigation and hand squeeze) plus a motor response task (actual hand squeeze). The timing of “action” instructions (expected motor response) and the actual motor response (P04 as an example) were used to determine the periods of responsiveness (PreLOR), loss of responsiveness (LOR), and recovery of responsiveness (ROR). Two 10-min resting-state fMRI, and two 15-min task fMRI (baseline) were studied before (Base1) and after (Base2) propofol infusion. (**b**) Probability maps of active regions across 5 subjects. The ROIs were individually defined for each condition by the overlapped activation maps across the four sessions, Base1, PreLOR, ROR and Base2. The ROIs included the supplementary motor area (SMA), premotor cortex (PreM), and precuneus/inferior parietal lobule (PreCu/IPL) for tennis imagery; the parahippocampal place area (PPA) and posterior parietal lobe (PPC) for navigation imagery; the SMA and PreM for squeeze imagery; and the primary motor cortex (M1) for actual motor response.

We studied brain signatures of mental imagery using task paradigms formerly applied to evaluate patients with disorders of consciousness. In addition to tennis and navigation imagery, a squeeze imagery task was included where volunteers were instructed to imagine squeezing an MRI compatible grip dynamometer (a rubber ball). Five volunteers were tested using functional MRI during and after stepwise propofol infusion. The infusion was controlled to achieve target effect-site concentrations from 0 to 2.4 μg/ml with each step (0.4 μg/ml) maintained for 5 minutes. After the highest dose, the infusion was terminated to allow spontaneous emergence. Overt behavioral responsiveness was assessed by motor response (squeezing a rubber ball by hand), which defined the periods during which a subject retained responsiveness (PreLOR), loss of responsiveness (LOR), and recovery of responsiveness (ROR). Two 15-min task baselines were studied before (Base1) and after (Base2) the propofol infusion (Fig. 1a).

Consistent with previous reports^6,7,20,21^, mental imagery and motor response tasks elicited reliable, robust, and statistically distinguishable patterns of activation in specific regions of the brain when subjects can overtly respond during Base1, PreLOR, ROR and Base2. These regions included supplementary motor area (SMA), premotor cortex (PreM) and precuneus/inferior parietal lobule (PreCu/IPL) during tennis imagery; the parahippocampal place area (PPA) and posterior parietal lobe (PPC) during navigation imagery; the SMA and PreM during squeeze imagery; and the primary motor cortex (M1) during actual motor response (Fig. 1b).

### Dissociation of behavioral and brain responsiveness

**Figure 2:**
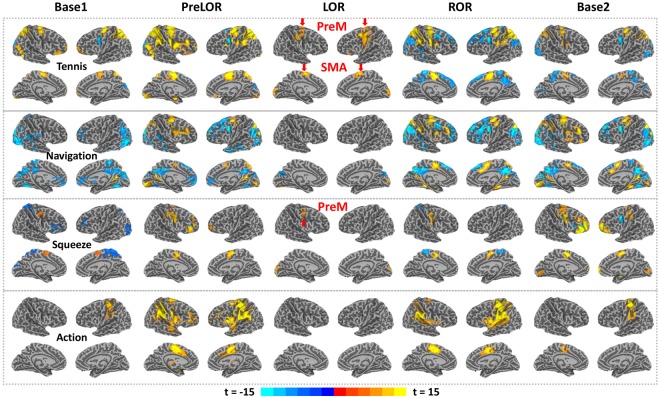
Significant brain activations during mental imagery and motor response tasks for one subject (P04). Whole brain activation maps are shown for tennis imagery, navigation imagery, squeeze imagery and motor response during five sessions: the first 15-min baseline task (Base1; no propofol infusion), the period before loss of responsiveness during propofol infusion (PreLOR), the period with loss of responsiveness (LOR), the period with recovery of responsiveness (ROR), and another 15-min baseline task (Base2; after recovery). Robust brain activations in SMA/PreM for tennis imagery and PreM for squeeze imagery during LOR were observed in this subject. All results were level-2 corrected at FPRs <5% of the individual level, with voxel-level p = 1.E-15 and a cluster size of 100 voxels (see Methods for more details).

We found robust brain activation in SMA/PreM for tennis imagery and PreM for squeeze imagery during LOR in one (P04) out of five studied subjects (Fig. 2), which confirmed our hypothesis. The results passed through rigorous control of false positive rates (FPRs) namely, level-2 correlation with FPRs <5% at the individual level (Supplementary Fig. 1 for the methodology demonstration, Supplementary Fig. 2 for a comparison without level-2 correction, and Supplementary Figs 3–6 for all subjects’ results after level-2 correction). Of note, the surviving threshold for SMA/PreM during tennis imagery for P04 was p = 1.E-26 with a cluster size of 100 voxels corresponding to FPRs = 0.001. In other words, if the result were false positive, we would only have observed one among 1000 subjects.

### Time-locked and delayed brain activity during behavioral unresponsiveness

**Figure 3:**
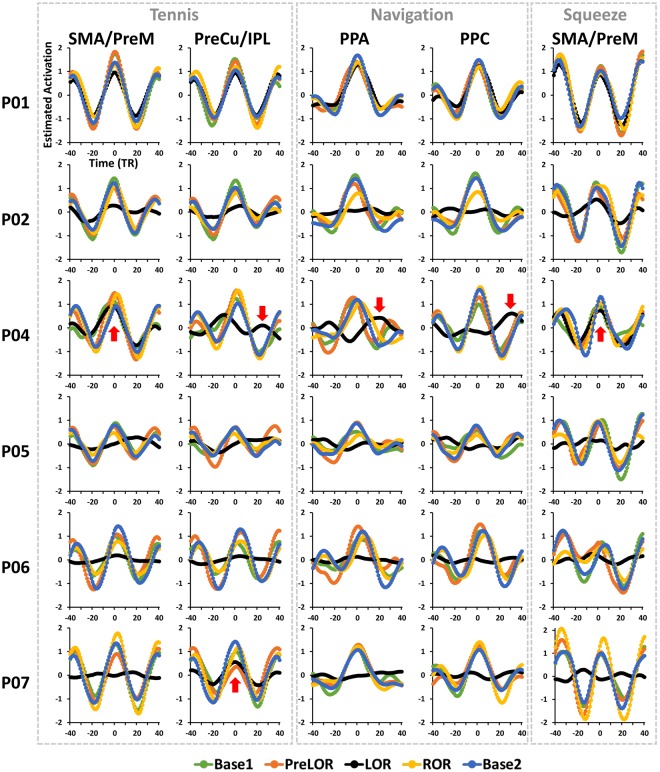
Time-locked and delayed brain responses during mental imagery tasks. To assess the effect of possible response time delay as a function condition, the events’ timing was shifted backward and forward up to −32 s (40 TRs; TR = 0.8 s) and +32 s in steps of 0.8 s (1 TR) centered on the instruction onset (0 s). The curves represent the estimated activation changing as a function of time shift shown for the ROIs of each imagery task across five sessions (Base1, PreLOR, LOR, ROR, and Base2). Results for subject P01 with saline (no propofol administration) served as a control. Note that the session segmentations of P01 were taken from that of P04, so that P01 was always responding.

For P04, we confirmed that the brain activity for tennis and squeeze imagery during LOR was indeed time-locked to the mental imagery instructions (peak amplitude at around t = 0 s) as it was during Base1, PreLOR, ROR and Base2 (Fig. 3). Interestingly, we observed a substantial time delay of brain activations in PreCu/IPL (peaking at +20.8 s after instruction onset; 26 TRs) during tennis imagery, PPA (peaking at +16.8 s; 21 TRs) and PPC (peaking at +24 s; 30 TRs) during navigation imagery for P04 (Fig. 3). The brain activations in the latter two regions at their corresponding peaks were significant by level-2 correction.

In addition, we found another subject (P07) who showed significant, time-locked brain activation in PreCu/IPL (level-2 corrected) during tennis imagery (Fig. 3 and Supplementary Fig. 3). However, unlike SMA/PreM, activation in PreCu/IPL alone may not be firm evidence for successfully tennis imagery.

Taken together, these results demonstrate that at least one subject in our sample retained the capacity for mental imagery as identified by fMRI, despite behavioral unresponsiveness during propofol sedation.

### Functional connectivity correlates of imagery vs. absent motor response

**Figure 4:**
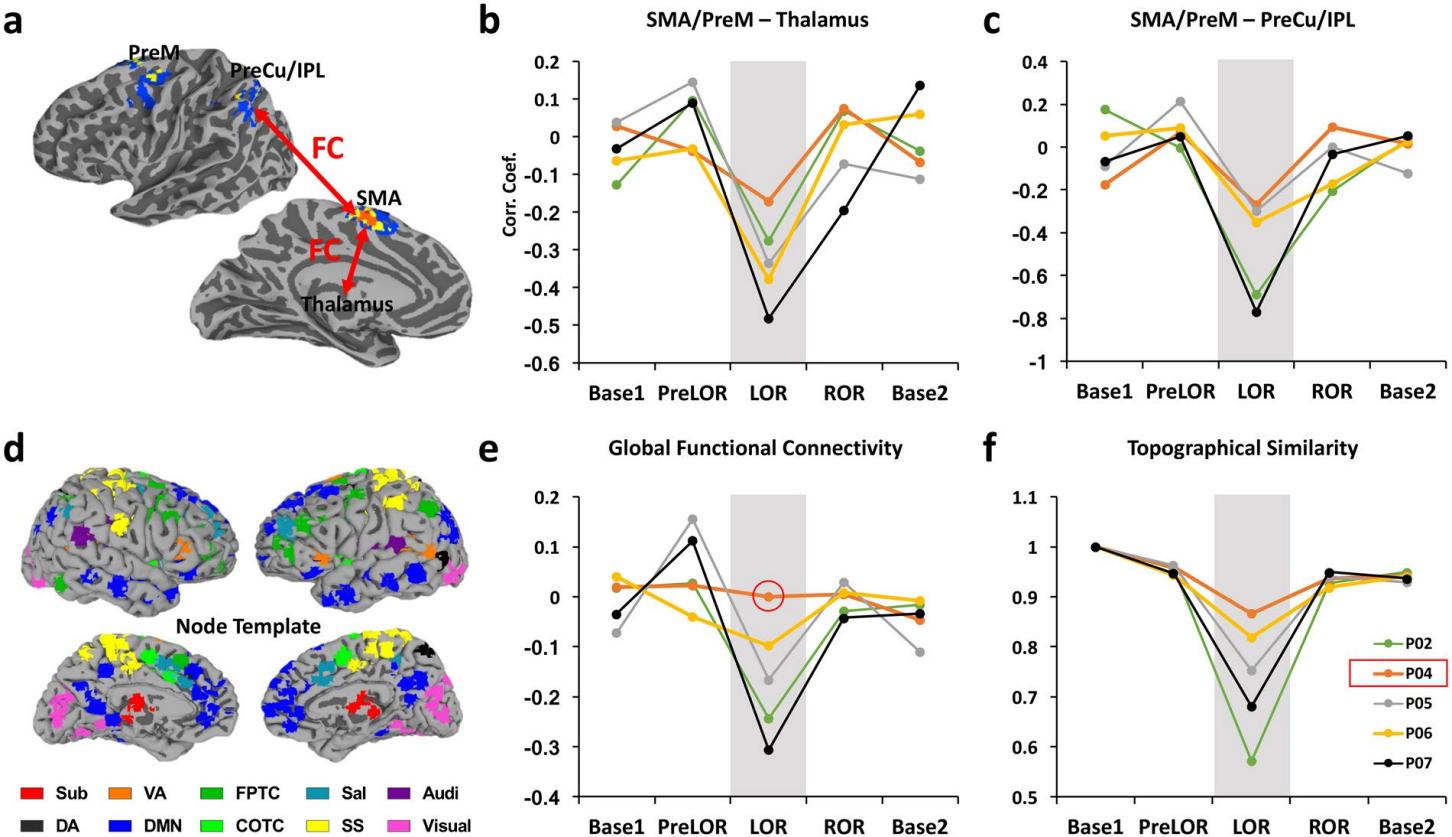
Functional connectivity correlates of task-response. (**a**) Schematic illustration of functional connectivity between pre-defined ROIs including SMA/PreM, thalamus and PreCu/IPL. (**b**) Disrupted functional communication of SMA/PreM-Thalamus during LOR at the group level: df = 4, F = 9.93, p = 0.00031, LOR < Base1 (p < 0.01), LOR < PreLOR (p < 0.01), LOR < ROR (p < 0.01), LOR < Base2 (p < 0.01). (**c**) Disrupted functional communications of SMA/PreM-PreCu/IPL during LOR at the group level: df = 4, F = 10.69, p = 0.00021, LOR < Base1 (p < 0.01), LOR < PreLOR (p < 0.01), LOR < ROR (p < 0.01), LOR < Base2 (p < 0.01). (**d**) A node template containing 226 brain areas within 10 functional networks including subcortical (Sub), dorsal attention (DA), ventral attention (VA), default mode (DMN), fronto-parietal task control (FPTC), cingulo-opercular task control (COTC), salience (Sal), sensory/somatomotor (SS), auditory (Audi), and visual networks (Visual). Pearson correlation coefficient of the time courses was computed between each pair of nodes. (**e**) Global functional connectivity (GFC) obtained as the mean FCs of all pairs of nodes. The GFC was significant reduced during LOR at the group level: df = 4, F = 5.87, p = 0.00419, LOR < Base1 (p < 0.05), LOR < PreLOR (p < 0.01), LOR < ROR (p < 0.05), LOR < Base2 (n.s.). (**f**) Topographical similarity (Topo) during LOR was significant reduced at the group level: df = 3, F = 15.06, p = 0.00023, LOR < PreLOR (p < 0.01), LOR < ROR (p < 0.01), LOR < Base2 (p < 0.01). Note that the GFC of P04 during LOR were almost unchanged, and its Topo was the least reduced in all subjects. Line graphs illustrate the deviation of FC in each subject from the respective mean across the overt responsive states (Base1, PreLOR, ROR and Base2).

We further explored the possible neural correlates of LOR in all participants as well as the unique brain responsiveness of P04 who evidenced preserved mental imagery during LOR. We hypothesized that disrupted functional communication between motor planning areas (SMA/PreM) and other central hubs of the brain was responsible for the failure of initiating an overt response^22,23^, and that preserved global functional connectivity^24^ may account for the retained capacity for mental imagery during LOR. We thus compared the functional connectivity (FC) between SMA/PreM and two central hubs of the brain (thalamus and PreCu/IPL), global functional connectivity (GFC), and global topographical similarity (Topo) across five sessions (Base1, PreLOR, LOR, ROR and Base2). In agreement with our hypotheses, we found a significant reduction of the FCs of SMA/PreM-Thalamus and SMA/PreM-PreCu/IPL during LOR when compared to other states at the group level. In contrast, the GFC of P04 during LOR was almost unchanged, and its Topo was the least reduced in all subjects, albeit both GFC and Topo during LOR were reduced at the group-level (Fig. 4).

## Discussion

Here we report, for the first time, the exceptional case of a healthy study participant who displayed the specific fMRI signatures of mental imagery in response to the investigator’s request while being behaviorally unresponsive due to sedation with the general anesthetic propofol. Contrasting this case with four other participants who failed to produce evidence of mental imagery, the unique finding of dissociation between consciousness and behavioral responsiveness in a healthy participant has important implications. First, it demonstrates the possibility of using anesthetics in a within-subjects study design to test methods and statistical paradigms of assessing covert consciousness in behaviorally nonresponsive patients in general. Second, our results suggest that structural brain damage is not a necessary condition for a detectable dissociation between consciousness and responsiveness to occur.

Successful mental imagery not only requires language comprehension, working memory, and decision making but also conscious effort to volitionally execute the task^7^. It is unlikely that imagery was due to an automatic evoked response or implicit preconscious neural response^15,25^. This is supported by the observation that activation in the imagery related regions (e.g. SMA and PPA) was absent when healthy participants listened to but failed to follow instructions^26^. Moreover, Cruse and colleagues found no appropriate EEG modulations in response to the instructions when participants were not actively following the instructions for a mental imagery task^9^. Collectively, our observations suggest that the sedated subject (i.e. P04) was conscious despite his failure to initiate an overt motor response. Moreover, mental imagery occurred without explicit recall, as confirmed by our post-scan inquiry, consistent with propofol’s amnesic effect^27,28^. Accepting this interpretation would imply that the transition between consciousness and unconsciousness may be more appropriately defined by the subject’s capacity for high-order cognitive tasks (e.g. mental imagery) rather than by initiating motor action.

Our functional connectivity data also support the above interpretation. In all tested subjects, functional connectivity between motor planning areas (SMA/PreM) and two central hubs of the brain (thalamus and PreCu/IPL) were disrupted during LOR. This functional disruption may be related to the failure of motor execution or forming intentions to motor actions^15,22,23^. On the other hand, the preservation of global connectivity of P04 during LOR suggests that the participant retained the overall functionality necessary for conscious processing in spite of the absence of a behavioral response.

Although P04 showed clear evidence of intentional neural modulation during tennis and squeeze imagery, the time course of navigation imagery was prolonged, together with reduced functional connectivity between a few central hubs of the brain. Therefore, subject P04 showed evidence of consciousness during LOR, but likely did not have the same phenomenological richness of normal waking consciousness. This may be analogous to patients who are emerging from anesthesia and can follow commands but have impoverished phenomenological content or the covertly aware VS/UWS patients who suffer serious global brain damage, possibly with memory impairments. Thus, it is not surprising that there are no accounts from recovered patients who report in a verifiable way on what it could have been like to be “conscious” in a vegetative state^15^.

The historical notion of voluntary motor imagery and motor execution was that they are partially overlapping processes that engage similar brain networks^29^. Hence, a reasonable argument could be made that if someone demonstrated evidence of consciousness via motor imagery that they should be able to engage intentional motor acts^13^. However, a recent study suggested that a possible biomarker for absent intentional movement in covertly aware patients was the selective structural damage to thalamocortical motor circuits^16^. In line with this proposition, we found that voluntary motor imagery and execution were dissociable (one could be present in the absence of the other). In addition, our results obtained in healthy volunteers take this further by showing that structural damage is not a necessary condition for a dissociation between consciousness and behavioral responsiveness. Accordingly, our findings favor the alternative account that intention might have a modular structure such that the subject’s ability to form intentions can be selectively impaired by the anesthetic without actual structural damage^15^. More specifically, we suggest that the intention to form mental imagery can be preserved while the intention to produce motor response is absent. This hypothesis may open new possibilities for detecting covert consciousness in VS/UWS patients in the absence of an identifiable structural damage to relevant motor circuits.

Although the number of participants scanned so far is insufficient to reliably estimate the general incidence of dissociation between behavioral and brain responsiveness, it is critical to note that this investigation was not designed as a typical neuroimaging study. The goal of this study was to demonstrate that *any* participant was capable of evidencing the dissociation of consciousness and behavioral responsiveness. Thus, like the original Owen *et al*.^6^ report on the dissociation between clinical assessment and covert consciousness in a single patient, or the report of Clauss *et al*.^30^ on zolpidem-induced recovery of consciousness in one semi-comatose patient, the identification of one such case using our protocol constitutes proof of principle that can form the basis for more systematic studies in the field.

The clinical validation of fMRI mental imagery tasks in detecting covert consciousness may still have a long way to go because rigorous validation would need to be based on an independent, objective measure of consciousness. To date, there is no “gold standard” measure of consciousness, thus neither the studies in neurological patients nor those using anesthesia may serve as a true validation. However, we take the view that the validity of a novel test for consciousness can nevertheless be supported by the degree to which its findings are consistent with a broad range of evidence from independent sources as well as other putative markers of consciousness^5,15,18,19^.

An earlier fMRI study by Davis and colleagues used a language-processing task during sedation with propofol in a within-subject design^31^. They tested methods of assessing covert cognitive abilities that were subsequently used in studying patients of disorders of consciousness^32,33^. Here, we advance this approach by assessing covert consciousness using fMRI mental imagery tasks while incrementally titrating the dose of propofol to and beyond loss of overt motor responsiveness. It is noteworthy that patients with VS/UWS and subjects under sedation/anesthesia exhibit some shared alterations of brain activity, such as globally reduced metabolic activity^34–36^, reduced functional connectivity^24,37–42^, and residual stimulus-evoked activities^31–33,43–45^. Therefore, we believe that the use of anesthetics in healthy participants will help accelerate the development of new approaches to detect covert consciousness in a rigorous and reproducible way that is difficult to achieve in the clinical setting for unresponsive patients with heterogeneous neuropathology.

## Methods

### Participants

The University of Michigan Institutional Review Board (IRB) approved the experimental protocol. All methods were performed in accordance with the relevant guidelines and regulations. Following written informed consent, seven subjects (ages between 22–30 years old; 4 males and 3 females) were recruited. Subjects were compensated for their participation. Strict confidentiality was maintained throughout. Subjects were assigned a code number following their first contact in the protocol. This number (e.g. P04) was used throughout the experiment and was the only identifier on specimen samples, behavioral and physiological archival data, and magnetic resonance (MR) scans. The first subject (P01) was studied with saline (no propofol administration) as a control subject. P03 was excluded from the analysis due to excessive head movement during fMRI scanning.

### Inclusion and exclusion criteria

Right-handed healthy participants of American Society of Anesthesiology physical status 1, aged 18–40, with a body mass index <30, who were experienced with racquet sports (at least 30 times over their lifetime) were eligible for inclusion. Subjects were excluded from participation if they did not speak English; had any contraindication to MRI scanning; possible pregnancy, extreme obesity, metallic substances in the body, claustrophobia, anxiety, or cardiopulmonary disease; or have an intracranial structural abnormality on T1-weighted MRI scans; or experience physical discomfort or excessive head movement during fMRI scanning. Potential subjects were also excluded if they had a history of neurological, cardiovascular, or pulmonary illness; significant head injury with loss of consciousness; learning disability or other developmental disorder; sleep apnea or any severe snoring history; or sensory/motor loss sufficient to interfere with performance of the study, gastroesophageal reflux disease, unwilling to abstain from alcohol use for 24 hours prior to their scheduled MRI study visit, history of drug use or a positive drug screen, tattoos on the head or neck region. Subjects with a history of allergic reaction to eggs are excluded.

### Anesthetic agents

Propofol was our reference drug because it has been the most widely-used agent in human fMRI studies of anesthetic effects and we have considerable experience in this field^24,28,38,46–49^. The advantage of propofol is that it exerts minimal effects on cerebral and systemic hemodynamics^50,51^ and therefore does not confound the fMRI results. Propofol suppresses neuronal activity mainly through an enhancement of GABA-A receptor-mediated inhibition thus modulating widespread targets throughout the brain.

### Anesthetic administration and monitoring

All subjects fasted for 8 hours before the study. An intavenous cannula was placed after a subcutaneous injection of lidocaine (1 ml of 1%) used as local anesthetic. Spontaneous respiration, end-tidal CO2, heart rate, and electrocardiogram were continuously monitored during the experiment. Noninvasive arterial pressure was measured with MR-compatible automatic monitor. Supplemental oxygen (2 L/min via nasal cannula) was used for all subjects. The propofol infusion rate was manually adjusted to achieve target effect-site concentrations (E.S.C.) of 0, 0.4, 0.8, 1.2, 1.6, 2.0, and 2.4 μg/ml as predicted by the Marsh TCI model^52^ with each step maintained for 5 minutes. After 5 minutes at the 2.4 μg/ml level, the propofol infusion was discontinued. Based on our previous studies^24,48^, the range of E.S.C. for loss of responsiveness (LOR) varies between 1.2 and 2.0 μg/ml. In order to obtain >10 min data of PreLOR for comparison, two increments (0.4 and 0.8 μg/ml) were added before 1.2 μg/ml. Another consideration was that a relatively low dose of propofol (E.S.C. close to LOR) often induces agitation, resulting in frequent or large head movements. To minimize head motion effects, we increased the final E.S.C. one step above the expected maximum for LOR to 2.4 μg/ml. In agreement with our previous studies^24,48^, the E.S.C. of LOR in our current studied sample (n = 5) ranged from 1.2 to 2.0 μg/ml (mean ± SD = 1.52 ± 0.33 μg/ml).

### Mental imagery and motor response tasks

Mental imagery and motor response tasks were studied before, during and after stepwise propofol infusion. Participants were asked to perform three imagery tasks (tennis, navigation and hand squeeze) plus a motor response task (actual hand squeeze). For tennis imagery, they were instructed to imagine standing still on a tennis court and to swing an arm to hit the ball back and forth to an imagined instructor. For navigation imagery, participants were instructed to imagine navigating the streets of a familiar city or to imagine walking from room to room in their home and to visualize all that they would see if they were there^6,7,20^. In the squeeze imagery task, participants were instructed to imagine squeezing an MRI compatible grip dynamometer (a rubber ball). In the motor response task, participants were instructed to actually grip the rubber ball (task following the squeeze imagery). A pseudo-randomized (Latin square) block design was applied, in which 15-second periods of tennis (and navigation) imagery, and 10-second periods of squeeze imagery with a hand squeeze within 5-second after hearing the instruction, alternated with 15-seconds of rest. The entire scan included 180 rest–imagery cycles (60 cycles per condition). The beginning of each trial was cued with the spoken word “tennis imagery”, “navigation imagery”, “squeeze imagery”, or “action”, and the rest period was cued with the word “relax”.

The verbal instructions were programmed using E-Prime 3.0 (Psychology Software Tools, Pittsburgh, PA) and delivered via an audiovisual stimulus presentation system designed for an MRI environment. The volume of the headphones was adjusted for subject comfort. Behavioral responses were measured in mmHg of air pressure during squeezing the rubber ball, using BIOPAC (https://www.biopac.com) MP160 system with AcqKnowledge software (V5.0). By comparing the timing of “action” instructions (expected motor response) and the actual motor response during and after stepwise propofol infusion, the periods during which a subject retained responsiveness (PreLOR), loss of responsiveness (LOR), and recovery of responsiveness (ROR) were determined. The offset of PreLOR, onset of LOR, offset of LOR, and onset of ROR were defined as the times of the last successful response of squeezing, the first failure to squeeze, the last failure to squeeze, and the first successful response of squeezing after LOR, respectively. The data during the transition periods between PreLOR and LOR and between LOR and ROR were not included in the analysis because the temporal resolution of the behavioral assessment (instruction to squeeze the ball) was 90 seconds on average, within which the behavioral responsiveness was uncertain.

### fMRI data acquisition

Data were acquired at University of Michigan Hospital using a 3T Philips scanner with a standard 32-channel transmit/receive head coil. Before fMRI scans, T1 weighted spoiled gradient recalled echo (SPGR) images was acquired for high spatial resolution of anatomical images with parameters: 170 sagittal slices, 1.0 mm thickness (no gap), TR = 8.1 s, TE = 3.7 ms, flip angle = 8°, FOV = 24 cm, image matrix 256 × 256 × 170. Functional images over the whole brain were acquired by a gradient-echo EPI pulse sequence with parameters: 21 slices, TR/TE = 800/25 ms by multiband acquisition, MB factor = 3, slice thickness = 6 mm, in-plane resolution = 3.4 × 3.4 mm; field of view (FOV) = 22 cm, flip angle = 76°, image matrix: 64 × 64. Participants were asked to lay at rest with eyes closed in the scanner for the first 10-min and the last 10-min resting-state scan. They were asked not to move and to stay awake. Verbal instructions were presented through earphones. Four task fMRI scans were conducted including 15-min wakeful baseline (Base1), during (30-min) and after (30-min) stepwise propofol infusion, and another 15-min recovery baseline (Base2).

### fMRI data preprocessing

Preprocessing steps were implemented in AFNI (http://afni.nimh.nih.gov/) including: (1) slice timing correction; (2) rigid body correction/realignment within and across runs; (3) coregistration with high-resolution anatomical images; (4) spatial normalization into Talaraich stereotactic space; (5) resampling to 3 × 3 × 3 mm^3^ voxels; (6) regressing out linear and nonlinear drift, head motion and its temporal derivative, mean time series from the white matter (WM) and cerebrospinal fluid (CSF) to control for physiological and non-neural noise, and band-pass filtered to 0.01–0.25 Hz; (7) spatial smoothing with 6 mm full-width at half-maximum isotropic Gaussian kernel; (8) the time-course per voxel of each run was normalized to zero mean and unit variance, accounting for differences in variance of non-neural origin (e.g., distance from head coil).

The issue of head motion artifacts was addressed rigorously based on prior studies^53,54^. Frame-wise displacement (FD) motion censoring was calculated using frame-wise Euclidean Norm (square root of the sum squares) of the six-dimension motion derivatives. A frame (TR) and its each previous frame were censored if the given frame’s derivative value has a Euclidean Norm above FD = 0.5 mm. A binary time series of motion censoring file per scan per subject was created and used in the following analyses.

### Individual subject general linear model (GLM) analyses

Four types of events corresponded to the three mental imagery tasks (tennis, navigation and squeeze), and actual motor response (action) were defined for each session. Events for each of these regressors were modeled and estimated by convolving onset times with a canonical hemodynamic response function using a BLOCK-model of the 3dDeconvolve function in AFNI. Estimated activation amplitude (beta) and t-statistics (two-sided) were calculated at the voxel level across the whole brain after censoring out the frames with FD >0.5 mm. This yielded four activation maps (tennis, navigation, squeeze and action) for each of the five sessions (Base1, PreLOR, LOR, ROR and Base2) per subject.

### Rigorous false positive control

The issue of inflated false-positive rates (FPRs) in fMRI analysis has drawn wide attention^55,56^. Several recommendations have been provided to control FPRs for group level analysis, such as setting primary voxel level p < 0.001 combined with estimated cluster size (number of voxels) to reach alpha <0.01 at the cluster level. As the primary focus of our study as well as others^6,7^ was to make statistical inference for a single subject, with potential clinical applications, it is crucial to control FPRs at the individual level^57^.

It can be expected that ongoing spontaneous BOLD signal fluctuations may coincide (to a certain extent) with the timing of task events simply by coincidence. These task-irrelevant activations may give rise false positive results, especially when limited number of trials were applied and when the frequency of task events lies in the dominant low frequency fluctuations of BOLD signal (0.01–0.1 Hz); for instance, in our current task design, there was approximately 30-second rest-task cycles (~0.03 Hz). We thus transformed the above issue by asking how much is the odds to observe positive results if the subjects were doing nothing (or mind wondering) during task-free state by applying the same task structure (e.g. task duration, inter-trial-internals, and number of trials).

First, we took the two 10-min resting state scans as task surrogate data and performed permutation-like analyses. We applied the same task structure (pseudo-event triggers) and shuffled the onset time (10 times) and the sequence (16 times) for the four conditions (tennis, navigation, squeeze and action), yielding 10 × 16 × 4 = 640 GLM analyses per subject. We applied this approach in 10 subjects, of which 7 subjects came from the current reported experiment and 3 subjects came from another experimental protocol with the same fMRI setting. This produced 6400 “activation” maps.

Second, we estimated the FPRs using AFNI’s upgraded function 3dClustSim that simulates noise volume assuming the spatial auto-correlation function is given by a mixed-model rather than a Gaussian-shaped function^56^. As a result, cluster level alpha <0.01 corresponds to voxel level p < 0.001 combined with a cluster size of 72–100 voxels; the range of cluster sizes was estimated based on the residual data of each GLM analysis across subjects. We then set voxel level p < 0.001 and the maximal cluster size of 100 voxels as a threshold (namely, level-1 correction) for the above 6400 maps. For a given map, if there was at least one cluster that survived at level-1 correlation, then it was considered as one positive observation. Activation (positive BOLD) and deactivation (negative BOLD) were counted separately. We next calculated the odds of positive observations (activation and deactivation) among the 6400 maps. Strikingly, the FPRs was 93% for activation and 95% for deactivation. Due to the high FPRs of the simulated results based on the resting-state data, the level-1 correction was obviously not appropriate for any statistical inference at the individual level.

We sought to reduce the FPRs by decreasing the voxel level p-values by fixing the cluster size at 100 voxels. In other words, we sought to delineate a distribution with FPRs change as function of voxel level p-values. This was achieved by threshholding the 6400 maps using multiple p-values ranging from p = 0.05 to p = 1.E-30. As a result, voxel level p = 1.E-15 with a cluster size of 100 voxels reduced the FPRs to <5%. We thereby defined this threshold as level-2 correlation for detecting “true” activation at the single-subject level.

### Definition of regions of interest (ROIs)

To control for individual anatomical variability, ROIs were defined for each condition (e.g. tennis) of each individual by the overlapped activation maps across the four sessions when subjects can overtly respond during Base1, PreLOR, ROR and Base2. Level-2 correlation was applied unless no activation was detected for a given condition/session (among the four)/subject; the voxel level p-value was then relaxed to one level higher (e.g. from p = 1.E-15 to 1.E-14) and so on until overlapped regions (cluster size >100 voxels) can be found. The imagery and motor response tasks have been shown to elicit reliable and distinguishable patterns of activation^6,7,20,21^. Based on these studies with the known neuroanatomical functions, our targeted ROIs were the supplementary motor area (SMA), premotor cortex (PreM) and precuneus/inferior parietal lobule (PreCu/IPL) for tennis imagery; the parahippocampal place area (PPA) and posterior parietal lobe (PPC) for navigation imagery; the SMA and PreM for squeeze imagery; and the primary motor cortex (M1) for actual motor response. Of note, there were individual variability of the ROIs during tennis and squeeze imagery. For instance, the SMA activation extended to PreM (forming a large cluster) during tennis imagery for some subjects, while either SMA or PreM was active during squeeze imagery for some subjects. To simplify the terminology, we referred SMA/PreM (a joint cluster, SMA or PreM alone) as the ROI name for tennis and squeeze imagery.

### Time-shifting analysis

To confirm that the mental imagery task-related brain activation was indeed time-locked to the instructions’ onset rather than potential signal contamination from the preceding trial, and to explore if there was any delayed brain activation due to the prolongation of intrinsic brain activity during sedation^24^, we performed the same GLM analysis but shifting the events timing backward and forward. Specifically, events for each regressor was shifted backward to −32 s (40 TRs; TR = 0.8 s) and forward to 32 s in steps of 0.8 s (1 TR) centered at the instruction onset (0 s). This yielded 81 activation maps for each condition (tennis, navigation, squeeze and action) of each session (Base1, PreLOR, LOR, ROR, and Base2) per subject. The estimated activation was then extracted for each time point from the above-defined ROIs. This produced curves with estimated activation changes as a function of time shift. If the brain activity were time-locked to the task, we expected to see a peak around t = 0 s. Alternatively, if there were delayed brain activity, the peak should have shifted forward.

### Functional connectivity between pre-defined ROIs

To investigate the changes in functional connectivity between brain regions (ROI-FC), the individual subjects’ mean BOLD signal time series from SMA/PreM (defined by squeeze imagery), bilateral thalamus (derived from the AFNI atlas), and PreCu/IPL (defined by tennis imagery) were extracted from each session. Qualitative estimation of functional connectivity changes was performed by calculating Pearson’s correlation coefficients (Fisher’s Z transformed) between SMA/PreM and thalamus, and between SMA/PreM and PreCu/IPL. Unlike conventional functional connectivity analysis applied in resting-state data, correlations between time series during tasks may indicate the integrity of functional interactions across regions with respect to the task^28,58–60^.

### Global functional connectivity

We adopted a well-established node template derived from a previous study^61^ and modified by our recent one^24^, containing 226 nodes (10 mm diameter spheres, 32 voxels per sphere) within 10 functional networks, namely, subcortical (Sub), dorsal attention (DA), ventral attention (VA), default mode (DMN), fronto-parietal task control (FPTC), cingulo-opercular task control (COTC), salience (Sal), sensory/somatomotor (SS), auditory (Audi), and visual networks (Visual). Next, we computed the Pearson correlation coefficient of the time courses (bandpass filtered to 0.01–0.1 Hz) between each pair of nodes, yielding a pairwise 226 × 226 correlation matrix (Fisher’s Z transformed) for each session of each subject. For a measure of global functional connectivity (GFC)^24^, we calculated the average of the triangular of the matrix of size 226 × (226-1)/2.

### Topographical similarity

Topographical similarity (Topo) was defined by the correlation coefficient between a reference correlation matrix (Base1) and a correlation matrix for other sessions (PreLOR, LOR, ROR and Base2) of each subject. Therefore, Topo quantifies the divergence of spatial connectivity configuration (among the nodes) from baseline^24^.

### Statistical analysis

Level-2 correction was applied for whole brain voxel-wise analysis, and statistical inference was made at the individual level. For the ROI-FC, GFC and Topo, repeated-measure ANOVAs with post-hoc Tukey HSD tests across five sessions were performed.

## Electronic supplementary material

Supplementary Information

## Data Availability

Original data that support the findings of this study are available from the corresponding authors (Z.H. and A.G.H.) upon reasonable request.
