# Supplementary material for: Brain imaging reveals covert consciousness during behavioral unresponsiveness induced by propofol
Source: Sci Rep. 2018 Sep 4;8:13195. doi: 10.1038/s41598-018-31436-z (PMC6123455; doi:10.1038/s41598-018-31436-z)
Supplement: Supplementary file 1 — Supplementary Information [file 41598_2018_31436_MOESM1_ESM.pdf]

Supplementary Information

Brain imaging reveals covert consciousness during behavioral unresponsiveness induced by propofol

Zirui Huang[1,2], Phillip E. Vlisides[1,2], Vijaykumar C. Tarnal[1,2], Ellen L. Janke[1,2], Kelley M. Keefe[1], Margaret M. Collins[1], Amy M. McKinney[1], Paul Picton[1], Richard E. Harris[1,2,3], George A. Mashour[1,2,3] & Anthony G. Hudetz[1,2,3]

[1]Department of Anesthesiology, University of Michigan Medical School, Ann Arbor, MI, USA

[2]Center for Consciousness Science, University of Michigan Medical School, Ann Arbor, MI, USA

[3]Neuroscience Graduate Program, University of Michigan, Ann Arbor, MI, USA

Correspondence

Z.H. (huangzu@umich.edu) and A.G.H. (ahudetz@med.umich.edu).

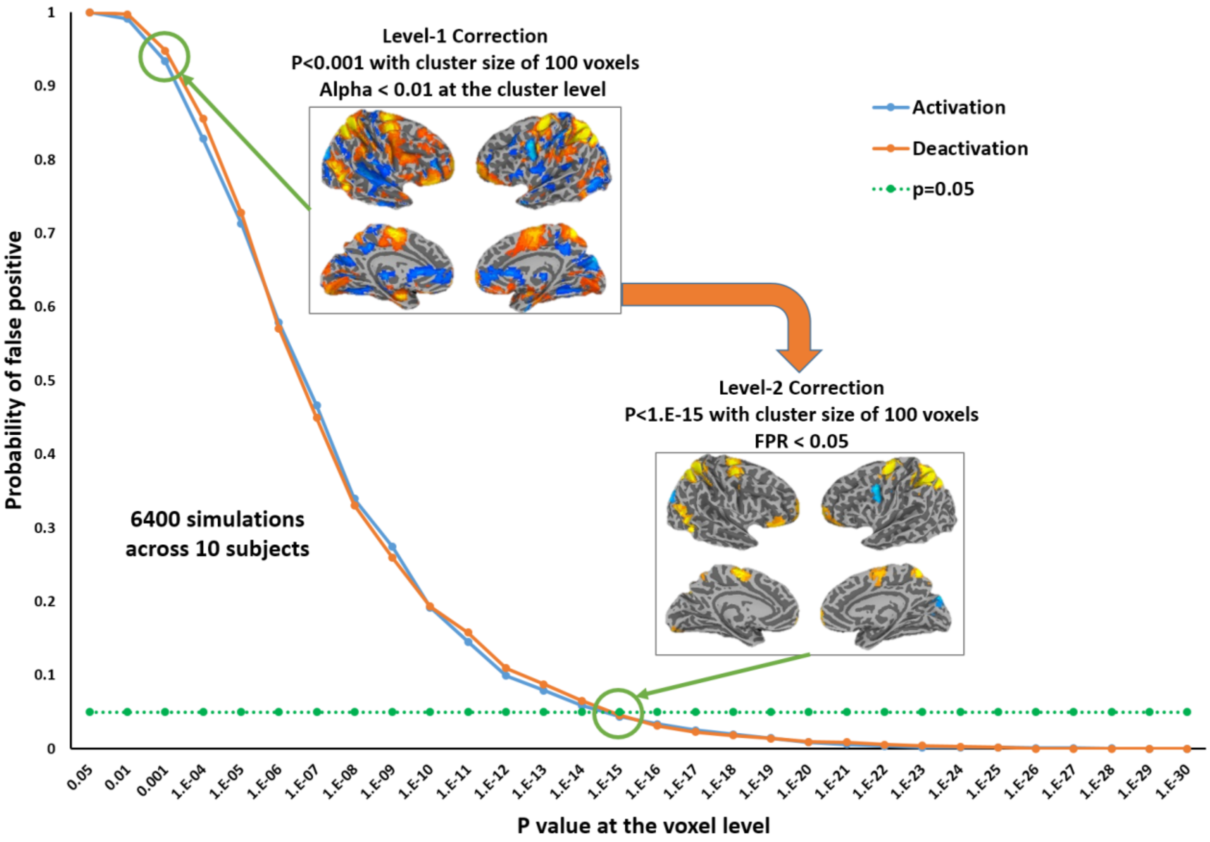

Supplementary Figure 1 | False positive rates (FPRs) estimation. 6400 simulations were performed using resting-state data with pseudo-event triggers. The FPRs were estimated as a function of voxel level p-values with fixed cluster size of 100 voxels. As a result, voxel level p=1.E-15 reduces the FPRs to <5%. This method is defined as level-2 correlation for detecting "true" activation at single-subject level.

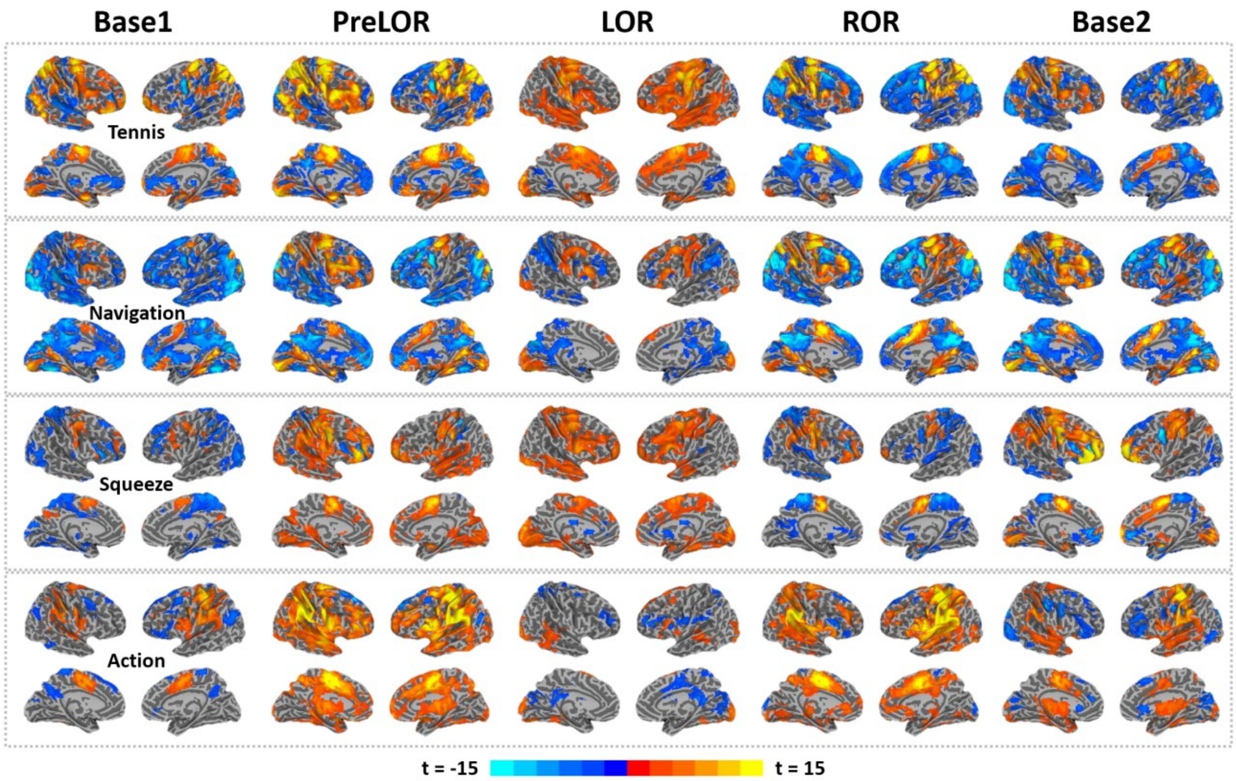

Supplementary Figure 2 | Brain activations for subject P04 using level-1 correction. Comparing to Figure 2 using level-2 correction, widespread brain activations were seen for tennis imagery, navigation imagery, squeeze imagery and motor response during all five sessions. Some of the activations may be false positive due to spontaneous activity.

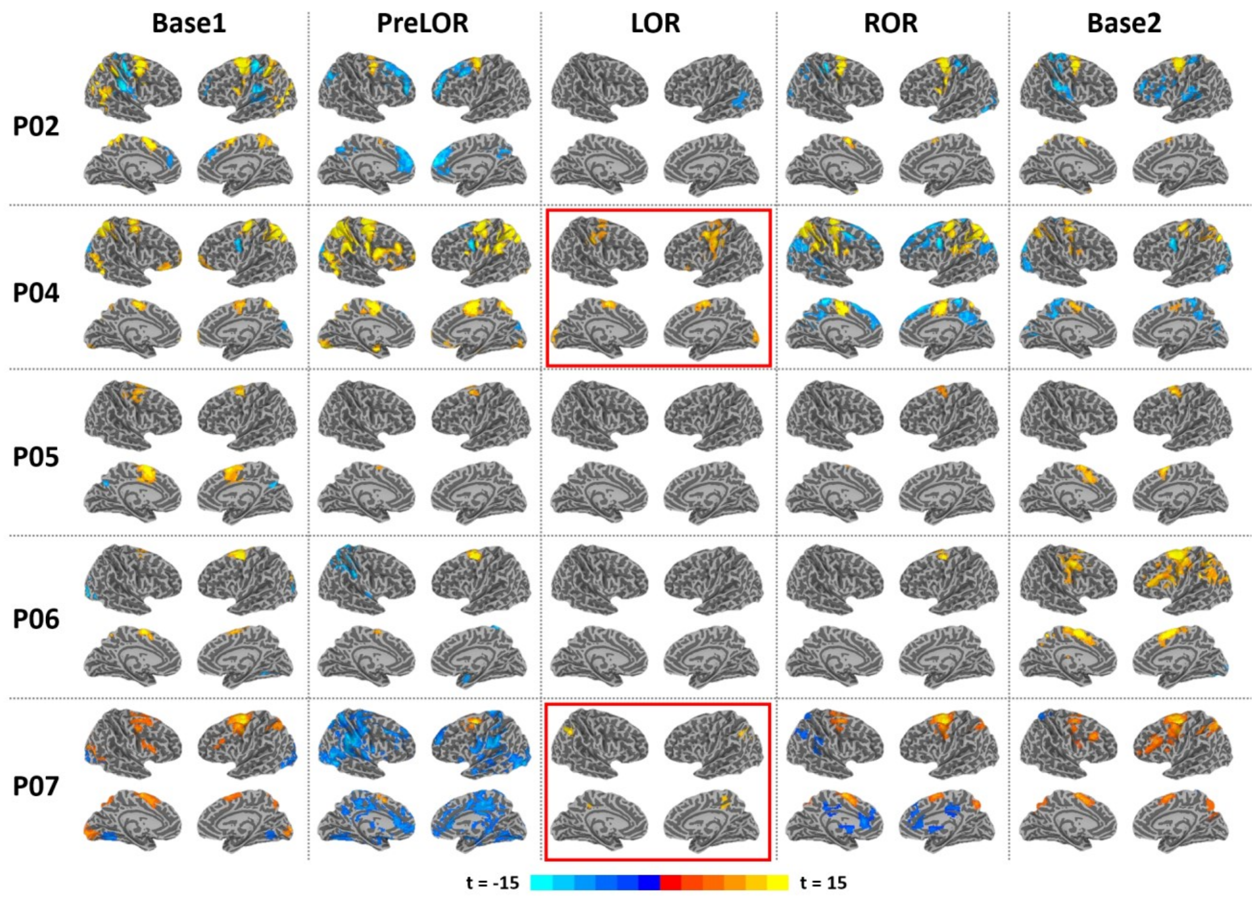

Supplementary Figure 3 | Significant brain activations during tennis imagery for all subjects. Robust brain activations in SMA/PreM and PreCu/IPL during LOR were observed in P04 and P07, respectively. All results were level-2 corrected with voxel-level p=1.E-15 and a cluster size of 100 voxels.

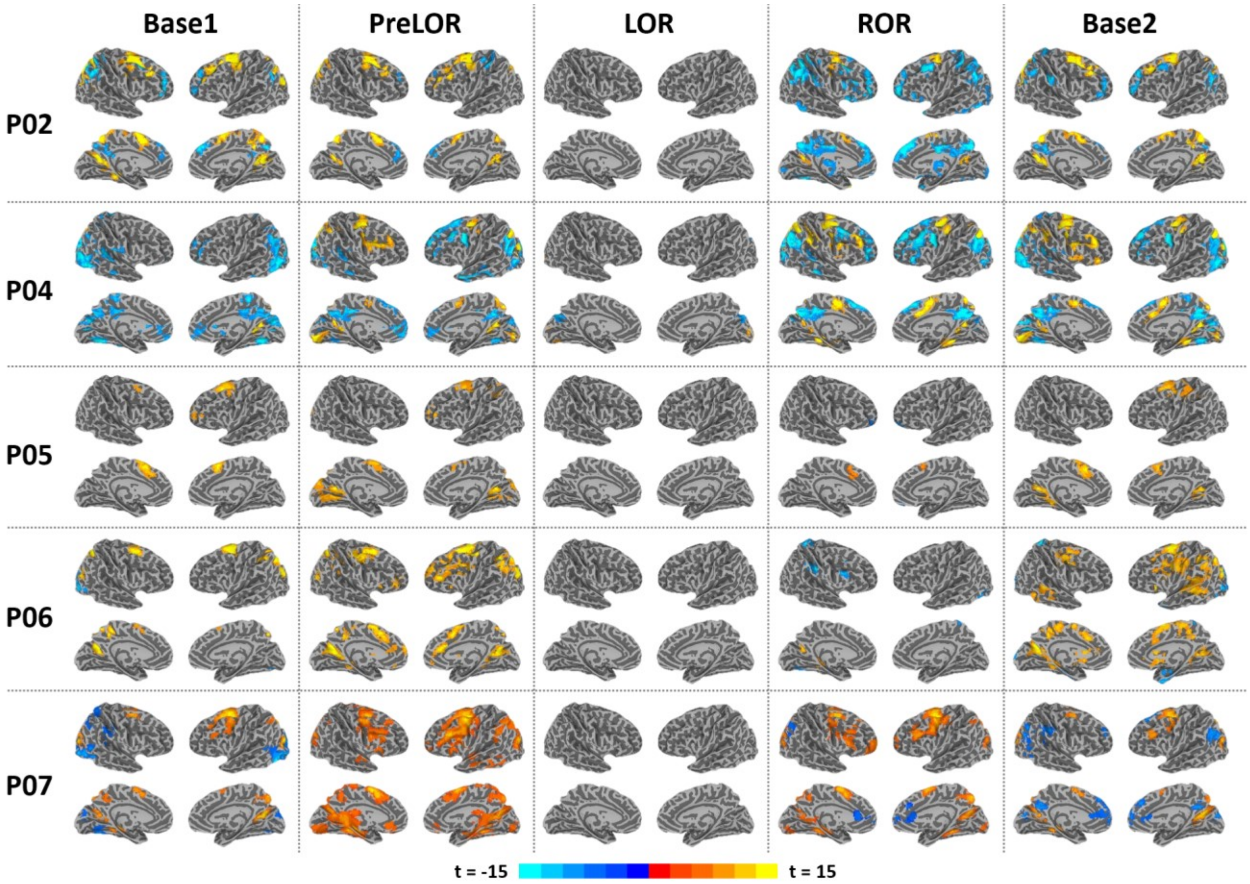

Supplementary Figure 4 | Significant brain activations during navigation imagery for all subjects. All results were level-2 corrected with voxel-level p=1.E-15 and a cluster size of 100 voxels.

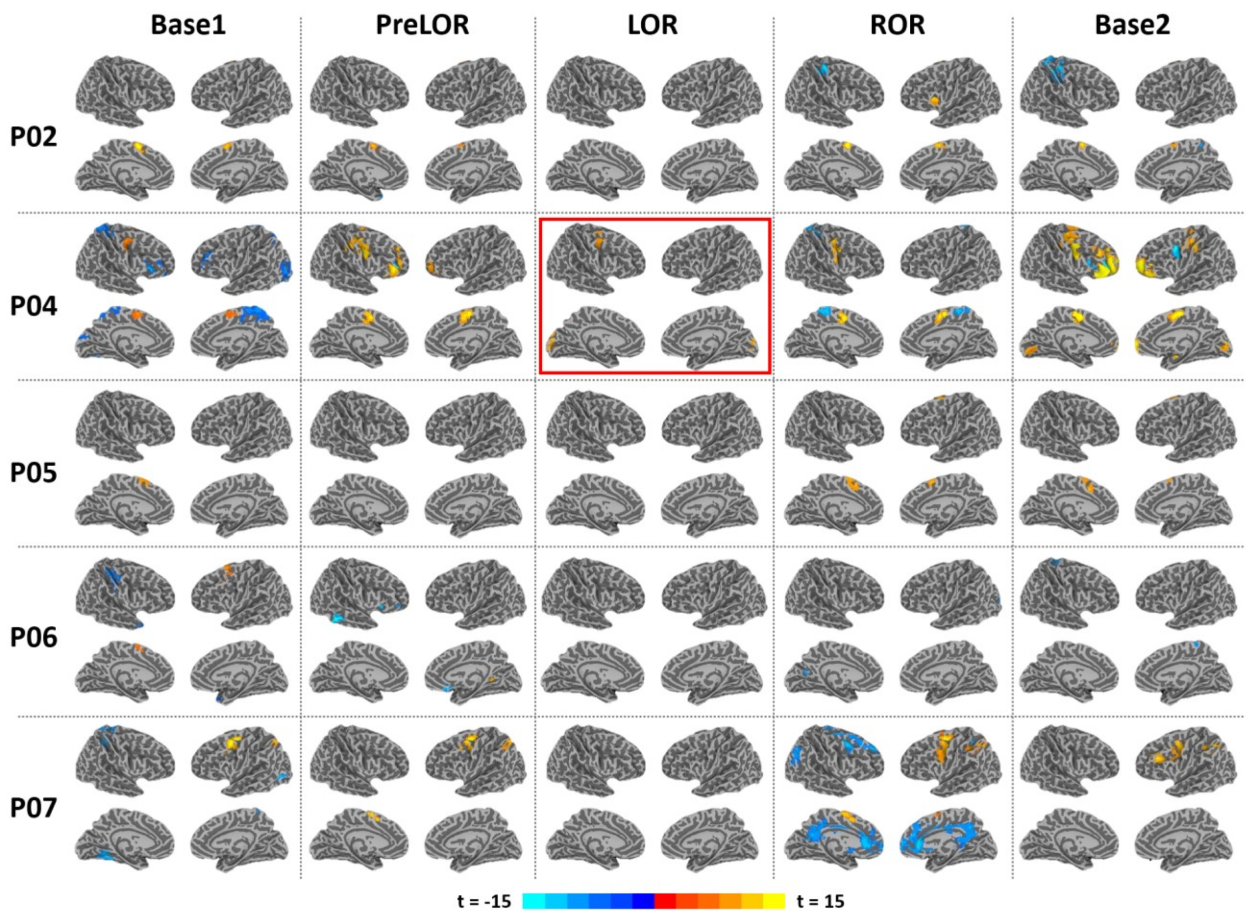

Supplementary Figure 5 | Significant brain activations during squeeze imagery for all subjects. Robust brain activations in PreM during LOR were observed in P04. All results were level-2 corrected with voxel-level p=1.E-15 and a cluster size of 100 voxels.

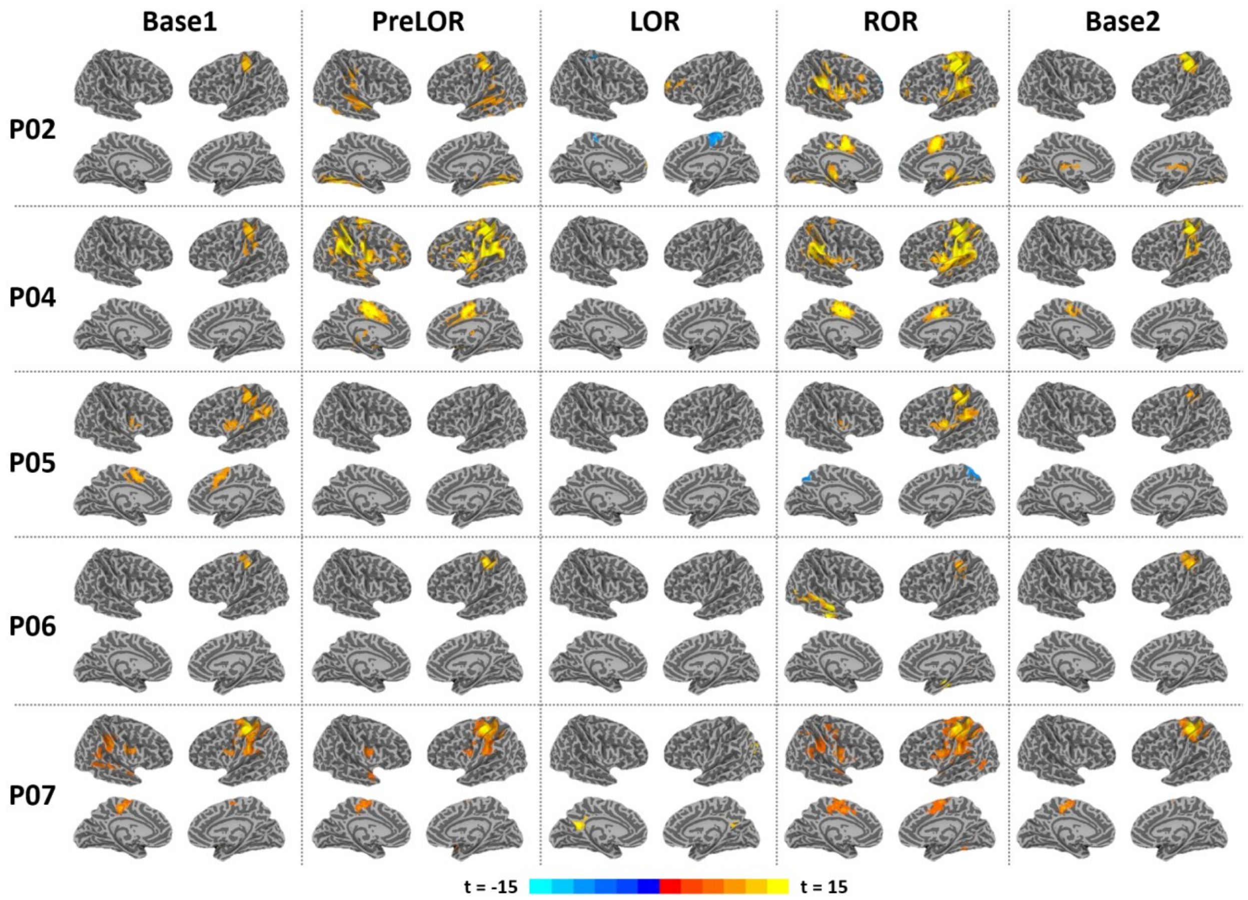

Supplementary Figure 6 | Significant brain activations during motor response for all subjects.

All results were level-2 corrected with voxel-level p=1.E-15 and a cluster size of 100 voxels.
